# Different associations between waist circumference and bone mineral density stratified by gender, age, and body mass index

**DOI:** 10.1186/s12891-022-05736-5

**Published:** 2022-08-17

**Authors:** Zhiqiang Yin, Haihong Yan, Yin Yu, Yupeng Liu

**Affiliations:** 1 Department of Orthopedics, Affiliated Zhongshan Hospital of Dalian University, Dalian, China; 2 Department of Cardiology, Affiliated Zhongshan Hospital of Dalian University, Dalian, China; 3 Administration Office, Affiliated Zhongshan Hospital of Dalian University, Dalian, China

**Keywords:** Waist circumference, Bone mineral density

## Abstract

**Introduction:**

Investigations of the relationship between waist circumference (WC) and bone mineral density (BMD) have inconsistent and incomprehensive results.

We explored the association between WC and BMD at various sites in a large-scale population-based study.

**Methods:**

We screened 5337 participants from National Health and Nutrition Examination Survey (NHANES) database. BMD was measured using dual-energy X-ray absorptiometry at various skeletal sites. The associations of WC with BMD were evaluated by weighted multivariable logistic regression models and conducted subgroup analyses for gender, age, and BMI. A weighted generalized additive model and a smooth curve fitting were performed to address non-linearity.

**Results:**

Adjustments for all confounders, in males, WC was negatively correlated to BMD in different age and BMI groups (all the *p* < 0.05), except for in the lowest BMI group; in females, overall trends of relationships between WC and BMD were negative. However, statistical differences were insignificant in some cases. Additionally, every 1 cm increase in WC for individuals of all ages with normal BMI (18.5 ≤ BMI < 25) was associated with decrease in BMD at each skeletal site, as was the case for men with BMI ≥ 25 kg/m^2^. For women, the negative association of WC with BMD was evident at the lumbar spine in the youngest age group (8 ≤ Age ≤ 18) with normal BMI.

**Conclusions:**

The nonlinear associations between WC and BMD at various skeletal sites are gender-, age- and BMI-specific in the NHANES (2006–2006).

**Supplementary Information:**

The online version contains supplementary material available at 10.1186/s12891-022-05736-5.

## Introduction

Osteoporosis is a metabolic and age-related skeletal disorder characterized by low bone mineral density (BMD) and microarchitectural deterioration, which increases bone fragility and susceptibility to fracture [1]. Along with the ageing social, the number of osteoporosis patients is rising, and osteoporosis-related fractures and secondary mortality are dramatically on the rise [1–3]. Unfortunately, the occurrence and progression of bone loss are usually silent, and patients have no symptoms until the first devastating fracture. Therefore, Therefore, it may be useful to explore simple anthtopometric risk factors for osteoporosis.

Accumulating epidemiological evidence has shown obesity is associated with.

osteoporosis. However, conclusions from different researcharches are conflicting. On the one hand, literature has reported that obesity, determined by body mass index (BMI), increases BMD and is protective against osteoporosis [4, 5]. In fact, however, almost half of all patients with osteoporosis-related fracturesture are overweight or obese [6]. On the other hand, body fat mass, especially abdominal fat mass, plays a detrimental role on BMD and the risk of fracture [7, 8]. One reason for this discrepancy might be the failure of BMI to serve as a sufficient biomarker of abdominal adiposity [9].

Waist circumference (WC) is suitable to assess abdominal adiposity and easy to standardize and clinically apply [10]. N evertheless, there few studies of the relations between WC and BMD are inconclusive [11]. The current study explores associations between WC and BMD at various skeletal sites in individuals stratified by age, gender, and BMI.

## Methods

### Study population

National Health and Nutrition Examination Survey (NHANES) is an extensive, ongoing cross-sectional survey conducted by the National Center for Health Statistics (NCHS). In detail, the NHANES database includes demographic, socioeconomic, dietary, and health-related data, examination components consisting of medical, dental, and physiological measurements, and laboratory tests administered by highly trained medical personnel. NHANES database can provide objective and overall data on health conditions for children and adults in the US, which is beneficial for researchers to develop sound public health policy and address emerging public health issues. All the data from the NHANES database were open and freely available for researchers throughout the world.

Our analysis was based on NHANES 2005–2006 data, a detailed flow chart was shown in Supplementary Fig. 1, and 5337 aged 8–69 years individuals were included in the final analysis ( dual-energy X-ray absorptiometry (DXA) was only administered to eligible participants aged 8–69 years in NHANES). Furthermore, individuals with age ≤ 18 are forbidden to smoke and drink in the united states, and physical activity is difficult to quantify. So data about smoking, alcohol use, and physical activity were unavailable in participants with less than or equal to 18.

### Variables

Waist circumference was regarded as an exposure variable in this study. Detailed measurement methods for WC are shown on the NHANES website (https://www.n.cdc.gov/nchs/data/nhanes/2005-2006/manuals/BM.pdf). Data about smoking, alcohol use, and physical activity were extracted from the questionnaire. Subjects who smoked less than 100 cigarettes in life were considered as never smokers. Former smokers were defined as having smoked over 100 cigarettes in their lifetime and as having quit smoking [12]. Alcohol use was categorized as follows [13]: lifetime abstainers < 12 drinks in entire life; former drinkers ≥ 12 drinks in the past but none during the past 12 months; for women, moderate: ≤ 1 drink per day; heavy: > 1 drink per day; for men: moderate: ≤ 2 drinks per day; heavy: > 2 drinks per day. Additionally, physical activity levels were classified by responding to the question "average level of physical activity each day” into sedentary, low, moderate and rigorous groups, respectively.

Continuous covariates included age, poverty income ratio, height, weight, alkaline phosphatase (ALP), total calcium, creatinine, fasting glucose, uric acid (UA), and parathyroid hormone. Race, smoking status, alcohol use, and physical activity were adjusted as categorical variables.

### Outcomes

The outcomes of the present study are BMD at various skeletal sites, including total body BMD, total femur BMD, femoral neck BMD, intertrochanteric BMD, lumbar spine BMD, and lumbar pelvis BMD. Dual-energy x-ray absorptiometry (DXA) is used in measurements of BMD, which is a widely accepted method of measuring BMD due to its speed, ease of use, and low radiation exposure. Importantly, the data of specific site BMD was from specific site DXA scans. Trained and certified radiology technologists performed the DXA examinations. Further details of the DXA examination protocol are documented in the Body Composition Procedures Manual on the NHANES website (https://www.cdc.gov/nchs/nhanes/index.htm).

### Statistical analysis

Continuous variables are reported as mean ± SD, and categorical variables are expressed as a number (percentage). All the statistical analysis in this study was conducted using package R version 3.4.3 (http://www.R-project.org) and EmpowerStats software (http://www.empowerstats.com). *p* < 0.05 is considered statistically significant. The associations of WC with BMD at various skeletal sites were evaluated by weighted multivariable logistic regression models. Two models were conducted: model 1: none was adjusted. Model 2: age, race, poverty income ratio, height, ALP, total calcium, creatinine, fasting glucose, UA, and parathyroid hormone were adjusted in group 1. Model 2: age, race, poverty income ratio, height, smoking status, alcohol use, physical activity, ALP, total calcium, creatinine, fasting glucose, UA, and parathyroid hormone were adjusted in group 2 and group 3. Subgroup analyses stratified by gender, age and BMI were also conducted. A weighted generalized additive model and a smooth curve fitting were deployed to address non-linearity.

## Results

### Participant characteristics

The demographic characteristics of 5337 participants in this study were demonstrated in Table 1 classified based on gender and age. No matter in male or female, significant differences were found among three age groups in race, poverty income ratio, height, weight, WC, smoking status, alcohol use, physical activity, ALP, total calcium, creatinine, fasting glucose, UA, and parathyroid hormone (all the *p* < 0.001). Group 2 of both genders had the highest BMD compared with those in groups 1 and 3, except for BMD at the lumbar spine of men. In addition, for both males and females, BMI and WC increased in group 3 (*p* < 0.001).

**Table 1:** Demographic characteristics of study participants

Variables	Male (*n* = 2773) Group 1 (*n* = 1178) (8 ≤ Age ≤ 18)	Group 2 (*n* = 1117) (18 < Age ≤ 50)	Group 3 (*n* = 478) (50 < Age ≤ 69)	*p*	Female (*n* = 2564) Group 1 (*n* = 1160) (8 ≤ Age ≤ 18)	Group 2 (*n* = 975) (18 < Age ≤ 50)	Group 3 (*n* = 429) (50 < Age ≤ 69)	*p*
Age, y	13.43 ± 3.03	33.35 ± 9.75	59.71 ± 5.44	< 0.001	13.28 ± 3.10	33.45 ± 9.79	59.53 ± 5.35	< 0.001
Race, n(%)				< 0.001				< 0.001
Mexcina	380 (32.26%)	291 (26.05%)	95 (19.87%)		384 (33.10%)	228 (23.38%)	83 (19.35%)	
Other Hispanic	31 (2.63%)	44 (3.94%)	8 (1.67%)		35 (3.02%)	39 (4.00%)	11 (2.56%)	
Non-Hispanic White	309 (26.23%)	475 (42.52%)	239 (50.00%)		302 (26.03%)	419 (42.97%)	220 (51.28%)	
Non-Hispanic Black	395 (33.53%)	269 (24.08%)	119 (24.90%)		376 (32.41%)	231 (23.69%)	99 (23.08%)	
Other	63 (5.35%)	38 (3.40%)	17 (3.56%)		63 (5.43%)	58 (5.95%)	16 (3.73%)	
Poverty income ratio	2.28 ± 1.49	2.64 ± 1.61	3.04 ± 1.60	< 0.001	2.18 ± 1.50	2.70 ± 1.62	2.95 ± 1.59	< 0.001
Smoking, n(%)				< 0.001				< 0.001
Never smoking	0 (0.00%)	503 (45.03%)	156 (32.64%)		0 (0.00%)	545 (55.90%)	239 (55.71%)	
Current smoking	0 (0.00%)	321 (28.74%)	137 (28.66%)		0 (0.00%)	207 (21.23%)	78 (18.18%)	
Quit smoking	0 (0.00%)	185 (16.56%)	185 (38.70%)		0 (0.00%)	125 (12.82%)	112 (26.11%)	
Data unavailable	1178 (100.00%)	108 (9.67%)	0 (0.00%)		1160 (100.00%)	98 (10.05%)	0 (0.00%)	
Physical activity, n(%)				< 0.001				< 0.001
Sedentary	61 (5.18%)	166 (14.86%)	102 (21.34%)		73 (6.29%)	210 (21.54%)	102 (23.78%)	
Low	170 (14.43%)	496 (44.40%)	236 (49.37%)		185 (15.95%)	550 (56.41%)	242 (56.41%)	
Moderate	95 (8.06%)	239 (21.40%)	92 (19.25%)		77 (6.64%)	178 (18.26%)	76 (17.72%)	
Vigorous	38 (3.23%)	215 (19.25%)	47 (9.83%)		3 (0.26%)	37 (3.79%)	9 (2.10%)	
Data unavailable	814 (69.10%)	1 (0.09%)	1 (0.21%)		822 (70.86%)	0 (0.00%)	0 (0.00%)	
Alcohol use, n(%)				< 0.001				< 0.001
Lifetime abstainers	0 (0.00%)	58 (5.19%)	24 (5.02%)		0 (0.00%)	139 (14.26%)	79 (18.41%)	
Former drinkers	0 (0.00%)	95 (8.50%)	65 (13.60%)		0 (0.00%)	161 (16.51%)	103 (24.01%)	
Moderate drinker	0 (0.00%)	286 (25.60%)	190 (39.75%)		0 (0.00%)	140 (14.36%)	90 (20.98%)	
Heavy drinker	0 (0.00%)	472 (42.26%)	114 (23.85%)		0 (0.00%)	355 (36.41%)	101 (23.54%)	
Data unavailable	1178 (100.00%)	206 (18.44%)	85 (17.78%)		1160 (100.00%)	180 (18.46%)	56 (13.05%)	
Laboratory examination								
ALP, U/L	194.81 ± 110.7	71.46 ± 28.48	73.62 ± 25.05	< 0.001	104.72 ± 61.29	63.77 ± 19.73	77.32 ± 22.79	< 0.001
Total calcium, mmol/L	2.44 ± 0.08	2.40 ± 0.08	2.37 ± 0.09	< 0.001	2.41 ± 0.07	2.36 ± 0.08	2.39 ± 0.10	< 0.001
Creatinine, μmmol/L	72.63 ± 15.27	88.17 ± 14.84	98.73 ± 80.20	< 0.001	62.72 ± 10.57	68.35 ± 16.71	76.73 ± 32.75	< 0.001
Glucose, mmol/L	4.90 ± 0.86	5.26 ± 1.53	6.25 ± 2.84	< 0.001	4.77 ± 0.55	5.05 ± 1.42	6.01 ± 2.52	< 0.001
UA, μmmol/L	322.59 ± 69.53	351.30 ± 68.93	355.52 ± 78.28	< 0.001	257.36 ± 49.97	264.74 ± 57.90	295.49 ± 74.79	< 0.001
Parathyroid, pg/mL	40.93 ± 27.08	38.81 ± 17.76	49.97 ± 37.29	< 0.001	41.18 ± 20.70	41.59 ± 19.74	47.92 ± 24.53	< 0.001
BMD, gm/cm^2^								
Total body BMD	1.03 ± 0.16	1.23 ± 0.11	1.20 ± 0.12	< 0.001	1.01 ± 0.14	1.15 ± 0.10	1.08 ± 0.11	< 0.001
Total femur BMD	0.94 ± 0.19	1.08 ± 0.14	1.02 ± 0.15	< 0.001	0.88 ± 0.16	0.97 ± 0.13	0.88 ± 0.14	< 0.001
Femoral neck BMD	0.87 ± 0.17	0.94 ± 0.15	0.84 ± 0.13	< 0.001	0.82 ± 0.16	0.87 ± 0.13	0.76 ± 0.13	< 0.001
Inter-trochante BMD	1.06 ± 0.23	1.27 ± 0.17	1.20 ± 0.17	< 0.001	1.00 ± 0.20	1.14 ± 0.15	1.05 ± 0.17	< 0.001
Lumbar spine BMD	0.87 ± 0.19	1.06 ± 0.15	1.07 ± 0.18	< 0.001	0.93 ± 0.18	1.07 ± 0.14	0.99 ± 0.17	< 0.001
Lumbar Pelvis BMD	1.14 ± 0.25	1.39 ± 0.19	1.31 ± 0.19	< 0.001	1.15 ± 0.22	1.31 ± 0.16	1.21 ± 0.16	< 0.001
Physical examination								
Weight, kg	59.23 ± 21.60	84.33 ± 16.16	85.81 ± 15.66	< 0.001	55.24 ± 18.49	72.11 ± 16.96	74.27 ± 16.16	< 0.001
Standing height, cm	160.95 ± 16.41	175.52 ± 7.82	174.54 ± 7.68	< 0.001	154.43 ± 12.11	162.15 ± 6.76	161.09 ± 6.94	< 0.001
BMI, kg/m^2^	22.19 ± 5.45	27.34 ± 4.76	28.08 ± 4.67	< 0.001	22.69 ± 5.77	27.41 ± 6.14	28.55 ± 5.58	< 0.001
Waist circumference, cm	77.22 ± 15.07	95.06 ± 13.03	102.02 ± 12.50	< 0.001	77.54 ± 14.29	90.35 ± 14.11	95.53 ± 13.43	< 0.001

Data were presented as mean ± SD or n (%)

*BMD* Body mineral density, *ALP* Alkaline phosphatase. *UA* Uric acid. *SD* Standard deviation

### Associations between WC and BMD were stratified by gender and age

The results of the multivariate regression analyses between WC and BMD categorized by gender and age were presented in Table 2. In males, WC was negatively correlated to BMD at various skeletal sites in different age groups after complete adjustments (all the *p* < 0.05). Moreover, compared with age group 2 and group 3, every 1 cm increase in WC resulted in the relatively most decrease in BMD at all skeletal sites in age group 1. Interestingly, the relationships between WC and BMD in females grouped by age were complicated. Firstly, significant inverse associations after adjusting for confounders were found at all skeletal sites apart from the lumbar pelvis in age group 1.

**Table 2:** Associations between waist circumference and BMD at various skeletal sites stratified by gender and age

	**Group 1** **(8 ≤ Age ≤ 18)**	**Group 2** **(18 < Age ≤ 50)**	**Group 3** **(50 < Age ≤ 69)**
**Male**			
**Total body BMD**			
Model 1	0.005 (0.004, 0.005) < 0.00001	0.000 (-0.000, 0.001) 0.12737	0.001 (-0.000, 0.002) 0.08698
Model 2	-0.007 (-0.008, -0.005) < 0.00001	-0.005 (-0.006, -0.003) < 0.00001	-0.006 (-0.008, -0.003) < 0.00001
**Total femur BMD**			
Model 1	0.006 (0.006, 0.007) < 0.00001	0.002 (0.002, 0.003) < 0.00001	0.003 (0.002, 0.004) < 0.00001
Model 2	-0.007 (-0.009, -0.005) < 0.00001	-0.005 (-0.007, -0.004) < 0.00001	-0.005 (-0.008, -0.002) 0.00100
**Femoral neck BMD**			
Model 1	0.006 (0.005, 0.006) < 0.00001	0.001 (0.001, 0.002) 0.00008	0.003 (0.002, 0.003) < 0.00001
Model 2	-0.007 (-0.009, -0.005) < 0.00001	-0.004 (-0.006, -0.003) < 0.00001	-0.004 (-0.007, -0.001) 0.00380
**Intertrochante BMD**			
Model 1	0.008 (0.007, 0.008) < 0.00001	0.003 (0.002, 0.004) < 0.00001	0.004 (0.003, 0.005) < 0.00001
Model 2	-0.008 (-0.010, -0.005) < 0.00001	-0.006 (-0.008, -0.004) < 0.00001	-0.005 (-0.008, -0.002) 0.00480
**Lumbar spine BMD**			
Model 1	0.004 (0.003, 0.005) < 0.00001	-0.001 (-0.001, 0.000) 0.06256	0.001 (-0.000, 0.002) 0.15855
Model 2	-0.008 (-0.010, -0.006) < 0.00001	-0.005 (-0.008, -0.003) < 0.00001	-0.007 (-0.010, -0.003) 0.00047
**Lumbar Pelvis BMD**			
Model 1	0.010 (0.009, 0.010) < 0.00001	0.004 (0.003, 0.005) < 0.00001	0.004 (0.002, 0.005) < 0.00001
Model 2	-0.008 (-0.010, -0.005) < 0.00001	-0.003 (-0.006, -0.001) 0.00782	-0.006 (-0.009, -0.002) 0.00305
**Female**			
**Total body BMD**			
Model 1	0.005 (0.004, 0.005) < 0.00001	0.001 (0.000, 0.001) 0.00022	0.001 (-0.000, 0.001) 0.10008
Model 2	-0.002 (-0.004, -0.001) 0.00011	-0.002 (-0.003, -0.001) 0.00191	-0.002 (-0.004, -0.001) 0.01101
**Total femur BMD**			
Model 1	0.007 (0.006, 0.007) < 0.00001	0.004 (0.003, 0.004) < 0.00001	0.004 (0.003, 0.004) < 0.00001
Model 2	-0.002 (-0.004, -0.001) 0.00416	-0.001 (-0.003, -0.000) 0.03400	-0.001 (-0.003, 0.001) 0.24056
**Femoral neck BMD**			
Model 1	0.007 (0.006, 0.007) < 0.00001	0.003 (0.003, 0.004) < 0.00001	0.003 (0.002, 0.004) < 0.00001
Model 2	-0.002 (-0.003, -0.000) 0.04569	-0.001 (-0.002, 0.000) 0.14362	-0.002 (-0.004, -0.000) 0.03048
**Intertrochante BMD**			
Model 1	0.008 (0.007, 0.009) < 0.00001	0.004 (0.004, 0.005) < 0.00001	0.004 (0.003, 0.005) < 0.00001
Model 2	-0.003 (-0.005, -0.001) 0.00826	-0.002 (-0.003, -0.000) 0.03423	-0.001 (-0.004, 0.002) 0.42544
**Lumbar spine BMD**			
Model 1	0.005 (0.004, 0.005) < 0.00001	-0.000 (-0.001, 0.001) 0.72778	0.001 (0.000, 0.003) 0.01707
Model 2	-0.004 (-0.006, -0.002) < 0.00001	-0.003 (-0.005, -0.002) 0.00003	-0.002 (-0.005, 0.001) 0.15839
**Lumbar Pelvis BMD**			
Model 1	0.009 (0.009, 0.010) < 0.00001	0.004 (0.004, 0.005) < 0.00001	0.002 (0.001, 0.003) 0.00008
Model 2	-0.001 (-0.003, 0.001) 0.57598	0.001 (-0.001, 0.003) 0.27937	-0.003 (-0.005, 0.000) 0.05703

All the results were shown by β (95%CI) and *p*

*BMD* Body mineral density, *CI* Confidence interval, *ALP* Alkaline phosphatase, *UA* Uric acid

Model 1: adjusted for none

Model 2: adjusted for age, race, poverty income ratio, height, weight, ALP, total calcium, creatinine, fasting glucose, UA, and parathyroid hormone in group 1

Model 2: adjusted for age, race, poverty income ratio, height, weight, smoking status, alcohol use, physical activity, ALP, total calcium, creatinine, fasting glucose, UA, and parathyroid hormone in group 2 and group 3

Furthermore, WC had relatively more importance on lumbar spine BMD in the age group 1 (β: -0.004, 95%CI: (-0.006, -0.002), *p* < 0.001). Secondly, in age group 2, WC was negatively related to total body BMD, total femur BMD, intertrochanteric BMD, and lumbar spine BMD, not femoral neck and pelvis BMD. Similarly, relatively more importance of WC on the lumbar spine was found (β: -0.003, 95%CI: (-0.005, -0.002), *p* < 0.001). Thirdly, WC was independently associated with total body BMD and femoral neck BMD in age group 3, not BMD at other sites.

**Fig. 1:**
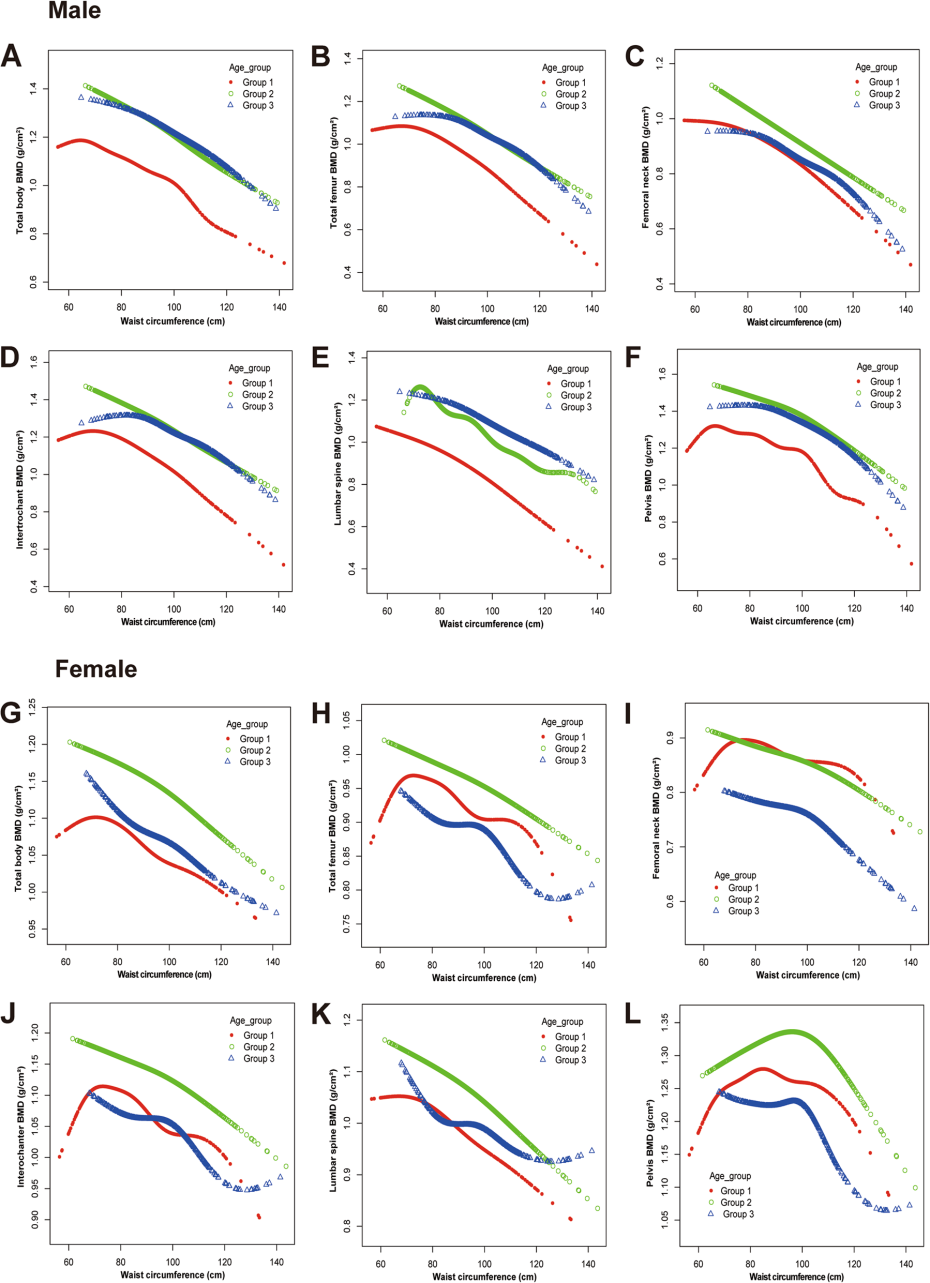
The association between WC and BMD stratified by gender and age. Group 1 (8 ≤ Age ≤ 18); Group 2 (18 < Age ≤ 50); Group 3(50 < Age ≤ 69). WC: waist circumference. BMD: body mineral density. ALP: alkaline phosphatase. UA: uric acid. Age, race, poverty income ratio, height, weight, ALP, total calcium, creatinine, fasting glucose, UA, and parathyroid hormone were adjusted in group 1. Further adjustments for smoking status, alcohol use, and physical activity were performed in group 2 and group 3

Smooth curve fittings and generalized additive models were used to characterize.

the nonlinear relationship between WC and BMD at various sites in individuals classified by gender and age is shown in Fig. 1. In men, among age groups 1 and 3, the association between WC and BMD was an inverted J-shaped except for lumbar spine BMD; among age group 2, the association between WC and lumbar spine BMD was an inverted J-shaped curve. In women, among the lowest age group, inverted U-shaped curves were presented between WC and BMD at different sites; among age group 2, inverted U-shaped curves owner was shown between WC and pelvis BMD; diverse curve types were illustrated in the highest age group. Nevertheless, the overall trend of BMD appeared to be downward with WC increasing.

### Associations between WC and BMD stratified by gender and BMI

The relationships between BMI and BMD at different sites were shown in supplementary table 1. In males, BMI was positively related to BMD even after adjustments for confounders (*p* < 0.001), except for BMD at the lumbar spine (β: 0.007; 95%CI: (-0.002, 0.016)). Meanwhile, BMI had a predominant and positive effect on BMD at the total femur, femoral neck, and intertrochanter (all the adjusted *p* < 0.05) in females. There were not dramatic relations between BMI and BMD at total body, lumbar spine and pelvis after full adjustments (β: 0.002, 95%CI: (-0.006, 0.009); β: 0.001, 95%CI: (-0.009, 0.012); β: 0.005, 95%CI: (-0.006, 0.017), respectively).

The results of the multivariate regression analyses between WC and BMD categorized by gender and BMI were presented in Table 3. Both in men and women, WC was not significantly related to BMD at various sites in BMI group 1 after adjusting for confounders (*p* > 0.05). In men, inverse and obvious associations between BMI groups 2 and 3 were demonstrated between WC and BMD at diverse skeletal sites after adjustments (*p* < 0.001). In women, WC had dramatically negative effects on BMD apart from BMD at the femoral neck and pelvis in model 2 in BMI groups 2 and 3.

**Table 3:** Associations between waist circumference and BMD at various skeletal sites stratified by gender and BMI

	**Group 1** **(BMI < 18.5)**	**Group 2** **(18.5 ≤ BMI < 25)**	**Group 3** **(BMI ≥ 25)**
**Male**			
**Total body BMD**			
Model 1	0.0134 (0.0119, 0.0150) < 0.0001	0.0049 (0.0037, 0.0060) < 0.0001	0.0006 (-0.0000, 0.0012) 0.0665
Model 2	-0.0018 (-0.0060, 0.0024) 0.4145	-0.0086 (-0.0103, -0.0069) < 0.0001	-0.0056 (-0.0067, -0.0044) < 0.0001
**Total femur BMD**			
Model 1	0.0097 (0.0078, 0.0115) < 0.0001	0.0010 (-0.0003, 0.0023) 0.1219	0.0012 (0.0005, 0.0020) 0.0007
Model 2	-0.0007 (-0.0063, 0.0049) 0.8064	-0.0101 (-0.0124, -0.0078) < 0.0001	-0.0059 (-0.0075, -0.0044) < 0.0001
**Femoral neck BMD**			
Model 1	0.0078 (0.0061, 0.0095) < 0.0001	-0.0015 (-0.0027, -0.0003) 0.0135	-0.0001 (-0.0008, 0.0007) 0.8728
Model 2	0.0028 (-0.0026, 0.0083) 0.3136	-0.0088 (-0.0110, -0.0065) < 0.0001	-0.0047 (-0.0062, -0.0032) < 0.0001
**Intertrochante BMD**			
Model 1	0.0126 (0.0104, 0.0148) < 0.0001	0.0023 (0.0008, 0.0038) 0.0025	0.0018 (0.0010, 0.0026) 0.0000
Model 2	-0.0012 (-0.0077, 0.0052) 0.7063	-0.0115 (-0.0143, -0.0088) < 0.0001	-0.0061 (-0.0078, -0.0043) < 0.0001
**Lumbar spine BMD**			
Model 1	0.0121 (0.0100, 0.0141) < 0.0001	0.0040 (0.0025, 0.0055) < 0.0001	0.0010 (0.0001, 0.0018) 0.0249
Model 2	-0.0026 (-0.0088, 0.0036) 0.4071	-0.0115 (-0.0140, -0.0090) < 0.0001	-0.0058 (-0.0076, -0.0041) < 0.0001
**Lumbar Pelvis BMD**			
Model 1	0.0173 (0.0152, 0.0194) < 0.0001	0.0051 (0.0035, 0.0066) < 0.0001	0.0018 (0.0008, 0.0028) 0.0003
Model 2	0.0013 (-0.0049, 0.0074) 0.6854	-0.0099 (-0.0127, -0.0071) < 0.0001	-0.0057 (-0.0077, -0.0036) < 0.0001
**Female**			
**Total body BMD**			
Model 1	0.0123 (0.0105, 0.0142) < 0.0001	0.0025 (0.0015, 0.0036) 0.0003	0.0010 (0.0004, 0.0016) 0.0007
Model 2	-0.0033 (-0.0085, 0.0019) 0.2148	-0.0020 (-0.0033, -0.0007) 0.0034	-0.0023 (-0.0033, -0.0014) 0.0002
**Total femur BMD**			
Model 1	0.0101 (0.0083, 0.0118) < 0.0001	0.0009 (-0.0002, 0.0020) 0.1252	0.0025 (0.0018, 0.0031) < 0.0001
Model 2	-0.0022 (-0.0083, 0.0040) 0.4944	-0.0020 (-0.0036, -0.0004) 0.0151	-0.0021 (-0.0033, -0.0009) 0.0009
**Femoral neck BMD**			
Model 1	0.0084 (0.0067, 0.0101) < 0.0001	-0.0003 (-0.0014, 0.0008) 0.6218	0.0017 (0.0010, 0.0024) 0.0002
Model 2	-0.0011 (-0.0074, 0.0053) 0.7472	-0.0010 (-0.0025, 0.0006) 0.2215	-0.0022 (-0.0034, -0.0010) 0.0332
**Intertrochante BMD**			
Model 1	0.0131 (0.0110, 0.0152) < 0.0001	0.0019 (0.0006, 0.0033) 0.0056	0.0029 (0.0021, 0.0037) < 0.0001
Model 2	-0.0003 (-0.0074, 0.0068) 0.9355	-0.0021 (-0.0040, -0.0002) 0.0347	-0.0024 (-0.0038, -0.0009) 0.0012
**Lumbar spine BMD**			
Model 1	0.0135 (0.0110, 0.0160) < 0.0001	0.0016 (0.0001, 0.0030) 0.0333	0.0004 (-0.0004, 0.0012) 0.3187
Model 2	-0.0030 (-0.0106, 0.0047) 0.4513	-0.0039 (-0.0058, -0.0020) 0.0001	-0.0033 (-0.0047, -0.0019) 0.0003
**Lumbar Pelvis BMD**			
Model 1	0.0178 (0.0154, 0.0202) < 0.0001	0.0046 (0.0031, 0.0061) < 0.0001	0.0021 (0.0013, 0.0030) 0.0001
Model 2	-0.0017 (-0.0099, 0.0065) 0.6855	-0.0004 (-0.0024, 0.0017) 0.7264	-0.0014 (-0.0029, 0.0001) 0.0771

All the results were shown by β (95%CI) and *p*

*BMD* Body mineral density, *BMI* Body mass index, *CI* Confidence interval, *ALP* Alkaline phosphatase, *UA* Uric acid

Model 1: adjusted for none

Model 2: adjusted for age, race, poverty income ratio, height, weight, smoking status, alcohol use, physical activity, ALP, total calcium, creatinine, fasting glucose, UA, and parathyroid hormone

**Fig. 2:**
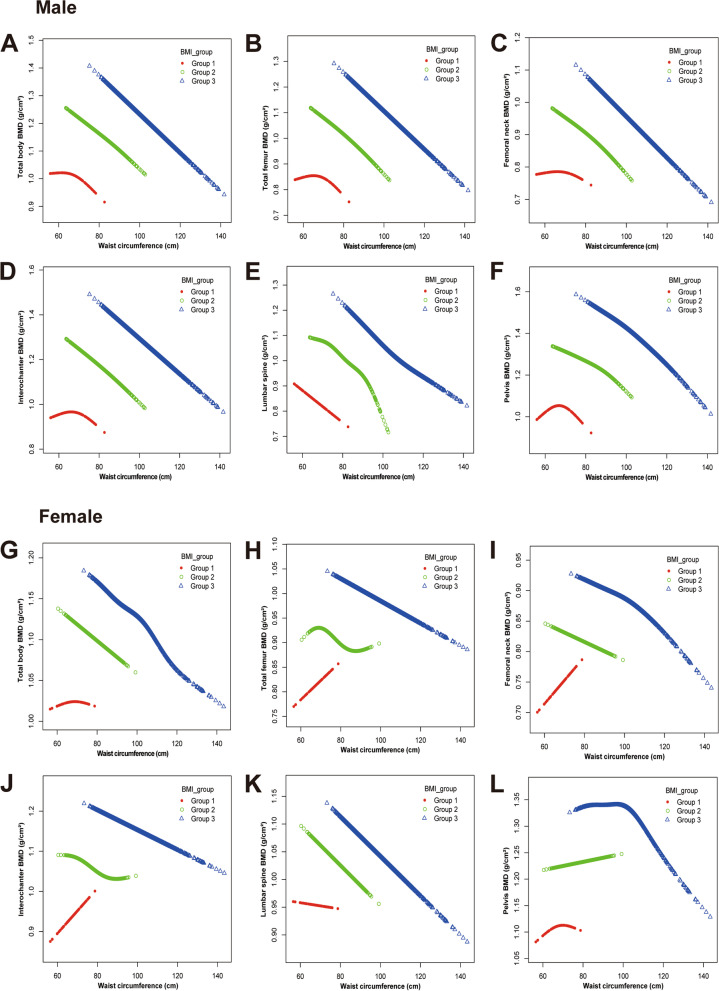
The association between WC and BMD stratified by gender and BMI. Group 1 (BMI < 18.5 kg/m^2^); Group 2 (18.5 kg/m^2^ ≤ BMI < 25 kg/m^2^); Group 3 (BMI ≥ 25 kg/m^2^). WC: waist circumference. BMD: body mineral density. BMI: body mass index. ALP: alkaline phosphatase. UA: uric acid. Age, race, poverty income ratio, height, weight, smoking status, alcohol use, physical activity, ALP, total calcium, creatinine, fasting glucose, UA, and, parathyroid hormone were adjusted

To detect nonlinear relationships between WC and BMD in subjects stratified by gender and BMI, Smooth curve fittings and generalized additive models were performed and related results were presented in Fig. 2. In males, WC had an inverted U-shaped relationship with BMD other than lumbar spine BMD in the lowest BMI group. In females, in the lowest BMI group, the associations between WC and total body BMD and pelvis BMD showed an inverted U-shaped curve; among BMI group 2, a light S-shaped association was presented between WC and total femur BMD and intertrochanter BMD; the association between WC and pelvis BMD was an inverted J-shaped curve in highest BMI group.

### The effect size of the association between WC and BMD according to gender, age, and BMI

**Fig. 3:**
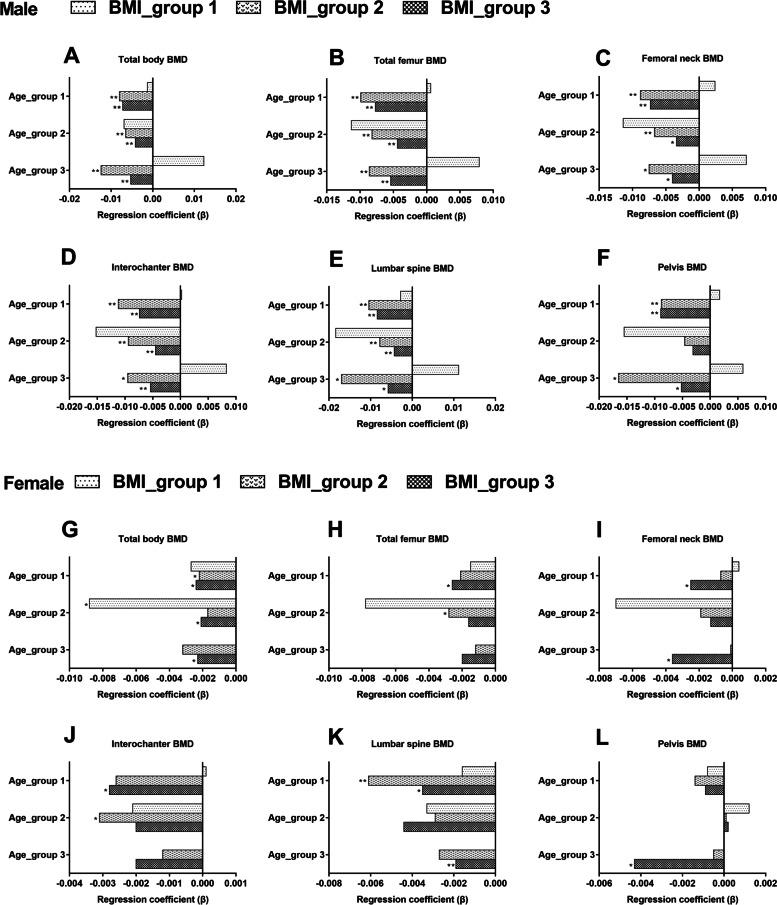
A comparison of effect size in the correlations between WC and BMD at different skeletal sites according to gender, age, and BMI. WC: waist circumference. BMD: body mineral density. BMI: body mass index. ALP: alkaline phosphatase. UA: uric acid. Age, race, poverty income ratio, height, weight, smoking status, alcohol use, medical activity, ALP, total calcium, creatinine, fasting glucose, UA, and parathyroid hormone were adjusted. *p < 0.05, ** *p* < 0.001

To define the relative importance of WC on bone health, we compared regression coefficients between body WC and BMD in subjects stratified by gender, age, and BMI (Fig. 3 and Supplementary Table 2). In men, based on the independent relations, every 1 cm increase in WC of individuals with normal BMI bought a relatively more decrease in BMD at any site in any age group than subjects with BMI ≥ 25 kg/m^2^. In women, due to the number of subjects with BMI ≥ 25 kg/m^2^ in age group 1 being zero, the related data is lacking. Moreover, the greatest effect of WC on BMD was presented at the lumbar spine in the lowest age group with normal BMI (β: -0.0061, 95%CI:(-0.0089, -0.0033), *p* < 0.001).

## Discussion

In this study, we explored the associations between waist circumference and BMD at various skeletal sites in subjects stratified by gender, age, and BMI. Our results indicated there were gender-, age- and BMI-specific relationships between WC and BMD. In males, waist circumference was significantly and inversely associated with BMD at all sites in all the age subgroups with normal BMI and overweight. WC is insignificantly related to BMD in subjects with low BMI in any age group at any site. Generally, WC has a negative effect on BMD in females. However, the differences were insignificant in some subgroups.

Several previous studies assessed the association between waist circumference as a metabolic syndrome component and BMD, but the results are inconsistent and uncomprehensive. A positive correlation between waist circumference and BMD was reported [14, 15]. Similarly, several studies found a negative correlation between WC and BMD [16, 17]. Furthermore, general population-based studies have found a significant negative correlation between BMD and waist circumference in postmenopausal females [18] and males [19]. The conflicting results may be attributed to the following factors. Different measuring methods were employed to evaluate BMD. Dual-energy x-ray absorptiometry (DXA) is a widely accepted and used method of measuring BMD. However, the ultrasound pulse transmission method was performed in some researches. Additionally, the number of screened individuals was small in some studies, and different studies focused on diverse populations and BMD at various skeletal sites. Most investigations paid more attention to older men and postmenopausal women and overlooked the relationships between WC and BMD in middle-aged people and adolescents. Furthermore, femoral neck fracture, secondary to decrease in femoral neck BMD, poses a major medical burden [20]. Thus, the researchers related femoral neck BMD attract more attention. Importantly, controlled covariates were diverse in different studies. Three studies presenting a positive correlation between waist circumference and BMD didn’t adjust body weight or BMI [14, 21]. Meanwhile, a significantly positive association was shown in several investigations. However, the association was negative after adjusting for body weight or BMI. From the above analysis, body weight or BMI may affect the association between WC and BMD. As reported in previous and present studies, greater body weight or BMI is thought to increase bone density, which may be attributed to an adaptive response of skeletons to growing loading. When the mechanical loading effect of body weight or BMI is statistically eliminated, fat mass, especially abdominal fat, may be negatively associated with bone health. In the present study, we assessed the relationships between WC and BMD at the various skeletal site and conducted subgroup analyses for gender, age, and BMI based on data from ongoing and large NHANES databases. At the same time, we adjusted all the cofounders, including age, race, poverty income ratio, height, weight, smoking status, alcohol use, physical activity, ALP, total calcium, creatinine, fasting glucose, UA, and parathyroid hormone, to present the real associations between WC and BMD. Fortunately, our study may be a contribution to filling the gap on this subject.

Different patterns of nonlinearity between WC and BMD were observed in different age groups in this study. This may be attributed to following several causes.

On the one hand, it is well known that bone metabolism dramatically changes with the growing of age. on the other hand, increased age can bring about changes of bone geometry, which is mainly manifested as bone expansion [22]. Furthermore, aging is associated with gradual changes in body composition, typically characterised by decreases in appendicular lean mass and increases in central fat mass [23, 24].

A cross-sectional study has shown total estradiol and free estradiol, but not testosterone levels were significantly correlated with BMD in males after various adjustments [21]; that is, estradiol may be a protective factor against bone loss, and variations in estradiol may have obvious effects on bone health. Additionally, testosterone predominates in males, and estrogen predominates in females. Therefore, sex hormones may play a more important role in females than in males, and adjustment for estrogen levels may be necessary for BMD-related studies, especially in females. In the present investigation, WC is significantly related to BMD at various sites in all age groups with normal BMI and overweight in males. However, the situation for females is a little more complicated. This may be because variations in estradiol are obvious among different age groups and attenuate the effects of WC on BMD.

An essential limitation of the present study is a cross-sectional design. The design allows only a cross-sectional observation of the associations of WC with BMD, so we fail to assess the effects of dynamic change of WC on BMD. Secondly, estradiol and variations in estradiol aren’t adjusted in statistical analysis. Further investigation should examine the link between WC and BMD under estradiol-adjusted conditions. Similarly, due to the unavailable exact age of menopause in the NHANES, the inclusion of this variable is lack in the study. Finally, data on overall diet quality and calcium intake are not available in this study.

## Supplementary Information

**Additional file 1: Supplementary Figure 1.** Study flow chart.

**Additional file 2: Table1.** The associations between BMI and BMD stratified by age and gender.

**Additional file 3: Table 2.** Associationsbetween waist circumference and BMD at various skeletal sites stratified by gender,age and BMI.

## Data Availability

The datasets analyzed during the current study are available in the website of the NHANES: https://www.cdc.gov/nchs/index.htm.
