# Supplementary material for: Different associations between waist circumference and bone mineral density stratified by gender, age, and body mass index
Source: BMC Musculoskelet Disord. 2022 Aug 17;23:786. doi: 10.1186/s12891-022-05736-5 (PMC9382731; doi:10.1186/s12891-022-05736-5)
Supplement: Supplementary file 1 — Additional file 1: Supplementary Figure 1. Study flow chart. [file 12891_2022_5736_MOESM1_ESM.docx]

**Supplementary Figure 1. Study flow chart.**

NHANESN, National Health and Nutrition Examination Survey.
