# Supplementary material for: Different associations between waist circumference and bone mineral density stratified by gender, age, and body mass index
Source: BMC Musculoskelet Disord. 2022 Aug 17;23:786. doi: 10.1186/s12891-022-05736-5 (PMC9382731; doi:10.1186/s12891-022-05736-5)
Supplement: Supplementary file 2 — Additional file 2: Table1. The associations between BMI and BMD stratified by age and gender. [file 12891_2022_5736_MOESM2_ESM.docx]

**Table 1 The associations between BMI and BMD stratified by age and gender.**

|  | **Group 1 (8≤Age≤18)** | **Group 2 (18<Age≤50)** | **Group 3 (50<Age≤69)** | **Total** |
| --- | --- | --- | --- | --- |
| **Male** |  |  |  |  |
| **Total body BMD** |  |  |  |  |
| Model 1 | 0.013 (0.011, 0.014)  <0.00001 | 0.003 (0.001, 0.004)  0.00009 | 0.004 (0.001, 0.006)  0.00188 | 0.008 (0.007, 0.009)  <0.00001 |
| Model 2 | -0.004 (-0.013, 0.005)  0.34049 | -0.011 (-0.025, 0.003)  0.12216 | 0.007 (-0.019, 0.034)  0.58665 | 0.007 (0.001, 0.014)  0.02655 |
| **Total femur BMD** |  |  |  |  |
| Model 1 | 0.018 (0.017, 0.020)  <0.00001 | 0.010 (0.009, 0.012)  <0.00001 | 0.012 (0.009, 0.014)  <0.00001 | 0.014 (0.013, 0.016)  <0.00001 |
| Model 2 | 0.001 (-0.011, 0.013)  0.87699 | -0.015 (-0.033, 0.003)  0.11126 | 0.037 (0.006, 0.068)  0.02073 | 0.017 (0.009, 0.025)  0.00005 |
| **Femoral neck BMD** |  |  |  |  |
| Model 1 | 0.017 (0.016, 0.019)  <0.00001 | 0.007 (0.006, 0.009)  <0.00001 | 0.009 (0.006, 0.011)  <0.00001 | 0.012 (0.011, 0.013)  <0.00001 |
| Model 2 | -0.005 (-0.017, 0.006)  0.36772 | -0.024 (-0.042, -0.006) 0.00982 | 0.031 (0.002, 0.060)  0.03399 | 0.011 (0.003, 0.018)  0.00941 |
| **Intertrochanter BMD** |  |  |  |  |
| Model 1 | 0.022 (0.020, 0.024)  <0.00001 | 0.013 (0.011, 0.014)  <0.00001 | 0.015 (0.011, 0.018)  <0.00001 | 0.018 (0.016, 0.019)  <0.00001 |
| Model 2 | 0.003 (-0.010, 0.017)  0.62463 | -0.014 (-0.036, 0.007)  0.18507 | 0.042 (0.005, 0.079)  0.02501 | 0.024 (0.014, 0.033)  <0.00001 |
| **Lumbar spine BMD** |  |  |  |  |
| Model 1 | 0.011 (0.009, 0.013)  <0.00001 | 0.000 (-0.002, 0.002)  0.84535 | 0.003 (0.000, 0.007)  0.04430 | 0.006 (0.005, 0.007)  <0.00001 |
| Model 2 | -0.006 (-0.017, 0.006)  0.32357 | -0.014 (-0.034, 0.007)  0.19262 | -0.006 (-0.047, 0.034)  0.76884 | 0.007 (-0.002, 0.016)  0.11848 |
| **Pelvis BMD** |  |  |  |  |
| Model 1 | 0.027 (0.025, 0.029)  <0.00001 | 0.015 (0.012, 0.017)  <0.00001 | 0.013 (0.010, 0.017)  <0.00001 | 0.020 (0.019, 0.022)  <0.00001 |
| Model 2 | -0.006 (-0.020, 0.008)  0.43202 | -0.015 (-0.039, 0.010)  0.25030 | 0.022 (-0.019, 0.062)  0.29457 | 0.016 (0.005, 0.026)  0.00383 |
| **Female** |  |  |  |  |
| **Total body BMD** |  |  |  |  |
| Model 1 | 0.012 (0.011, 0.013)  <0.00001 | 0.002 (0.001, 0.003)  <0.00001 | 0.003 (0.001, 0.004)  0.00947 | 0.007 (0.006, 0.007)  <0.00001 |
| Model 2 | 0.007 (-0.004, 0.017)  0.21898 | -0.004 (-0.016, 0.009)  0.56794 | -0.005 (-0.027, 0.017)  0.65136 | 0.002 (-0.006, 0.009)  0.61799 |
| **Total femur BMD** |  |  |  |  |
| Model 1 | 0.018 (0.016, 0.019)  <0.00001 | 0.010 (0.009, 0.011)  <0.00001 | 0.010 (0.008, 0.012)  <0.00001 | 0.013 (0.012, 0.014)  <0.00001 |
| Model 2 | 0.016 (0.001, 0.031)  0.03571 | 0.007 (-0.009, 0.022)  0.39799 | 0.015 (-0.011, 0.042)  0.25936 | 0.015 (0.006, 0.025)  0.00138 |
| **Femoral neck BMD** |  |  |  |  |
| Model 1 | 0.017 (0.016, 0.019)  <0.00001 | 0.009 (0.008, 0.010)  <0.00001 | 0.008 (0.006, 0.010)  <0.00001 | 0.013 (0.012, 0.013)  <0.00001 |
| Model 2 | 0.018 (0.003, 0.033)  0.01563 | 0.012 (-0.003, 0.026)  0.12431 | 0.007 (-0.017, 0.031)  0.55701 | 0.017 (0.008, 0.026)  0.00031 |
| **Intertrochanter BMD** |  |  |  |  |
| Model 1 | 0.021 (0.019, 0.022)  <0.00001 | 0.011 (0.010, 0.012)  <0.00001 | 0.012 (0.009, 0.014)  <0.00001 | 0.015 (0.014, 0.016)  <0.00001 |
| Model 2 | 0.014 (-0.003, 0.031)  0.11189 | 0.005 (-0.013, 0.023)  0.57341 | 0.024 (-0.008, 0.056)  0.13574 | 0.017 (0.005, 0.028)  0.00359 |
| **Lumbar spine BMD** |  |  |  |  |
| Model 1 | 0.012 (0.011, 0.014)  <0.00001 | 0.001 (-0.001, 0.002)  0.49364 | 0.004 (0.001, 0.007)  0.00589 | 0.006 (0.005, 0.007)  <0.00001 |
| Model 2 | 0.017 (0.002, 0.032)  0.02576 | 0.001 (-0.017, 0.019)  0.92686 | -0.039 (-0.072, -0.006)  0.02105 | 0.001 (-0.009, 0.012)  0.79727 |
| **Pelvis BMD** |  |  |  |  |
| Model 1 | 0.023 (0.021, 0.025)  <0.00001 | 0.010 (0.008, 0.011)  <0.00001 | 0.007 (0.004, 0.010)  <0.00001 | 0.015 (0.014, 0.016)  <0.00001 |
| Model 2 | 0.017 (-0.001, 0.035)  0.06762 | -0.010 (-0.030, 0.009)  0.30626 | -0.011 (-0.043, 0.020)  0.48339 | 0.005 (-0.006, 0.017)  0.36831 |

All the results were showed by β (95%CI) and *p.*

BMI: body mass index; BMD: body mineral density. CI: confidence interval. ALP: alkaline phosphatase. UA: uric acid.

Model 1: adjusted for none.

Model 2: adjusted for age, race, poverty income ratio, height, weight, smoking status, alcohol use, physical activity, ALP, total calcium, creatinine, fasting glucose, UA and parathyroid hormone.
