# Supplementary material for: Different associations between waist circumference and bone mineral density stratified by gender, age, and body mass index
Source: BMC Musculoskelet Disord. 2022 Aug 17;23:786. doi: 10.1186/s12891-022-05736-5 (PMC9382731; doi:10.1186/s12891-022-05736-5)
Supplement: Supplementary file 3 — Additional file 3: Table 2. Associationsbetween waist circumference and BMD at various skeletal sites stratified by gender,age and BMI. [file 12891_2022_5736_MOESM3_ESM.docx]

**Table 2 Associations between waist circumference and BMD at various skeletal sites stratified by gender, age and BMI.**

|  | | AGE_GROUP= 1 | AGE_GROUP= 2 | AGE_GROUP= 3 |
| --- | --- | --- | --- | --- |
| **Male** | |  |  |  |
| **BMI_GROUP= 1** | |  |  |  |
| **Total body BMD** | |  |  |  |
| Model 1 | | 0.0110 (0.0093, 0.0127) <0.000001 | -0.0013 (-0.0098, 0.0072) 0.775750 | 0.0158 (-0.0015, 0.0330) 0.116674 |
| Model 2 | | -0.0013 (-0.0054, 0.0029) 0.550369 | -0.0960 (, ) NaN | 0.1239 (, ) NaN |
| **Total femur BMD** | |  |  |  |
| Model 1 | | 0.0092 (0.0072, 0.0113) <0.000001 | -0.0006 (-0.0135, 0.0123) 0.926964 | 0.0264 (0.0025, 0.0504) 0.067306 |
| Model 2 | | 0.0006 (-0.0051, 0.0062) 0.844965 | -0.1127 (, ) NaN | 0.0788 (, ) NaN |
| **Femoral neck BMD** | |  |  |  |
| Model 1 | | 0.0084 (0.0065, 0.0103) <0.000001 | -0.0066 (-0.0188, 0.0056) 0.308182 | 0.0242 (0.0101, 0.0383) 0.012139 |
| Model 2 | | 0.0024 (-0.0034, 0.0082) 0.422005 | -0.1104 (, ) NaN | 0.0709 (, ) NaN |
| **Intertrochante BMD** | |  |  |  |
| Model 1 | | 0.0115 (0.0091, 0.0140) <0.000001 | 0.0018 (-0.0148, 0.0184) 0.834135 | 0.0308 (0.0030, 0.0587) 0.066836 |
| Model 2 | | 0.0002 (-0.0062, 0.0067) 0.946171 | -0.1516 (, ) NaN | 0.0827 (, ) NaN |
| **Lumbar spine BMD** | |  |  |  |
| Model 1 | | 0.0085 (0.0064, 0.0106) <0.000001 | -0.0033 (-0.0208, 0.0142) 0.716606 | 0.0176 (-0.0066, 0.0419) 0.197289 |
| Model 2 | | -0.0028 (-0.0089, 0.0033) 0.367873 | -0.1841 (, ) NaN | 0.1125 (, ) NaN |
| **Lumbar Pelvis BMD** | |  |  |  |
| Model 1 | | 0.0169 (0.0145, 0.0193) <0.000001 | -0.0050 (-0.0190, 0.0090) 0.494997 | 0.0212 (-0.0029, 0.0452) 0.128106 |
| Model 2 | | 0.0017 (-0.0047, 0.0080) 0.609433 | -0.1546 (, ) NaN | 0.0589 (, ) NaN |
| **BMI_GROUP= 2** | |  |  |  |
| **Total body BMD** | |  |  |  |
| Model 1 | | 0.0022 (0.0002, 0.0043) 0.031051 | -0.0012 (-0.0028, 0.0004) 0.144096 | -0.0014 (-0.0049, 0.0021) 0.441246 |
| Model 2 | | -0.0080 (-0.0104, -0.0055) <0.000001 | -0.0065 (-0.0093, -0.0038) 0.000004 | -0.0124 (-0.0185, -0.0062) 0.000191 |
| **Total femur BMD** | |  |  |  |
| Model 1 | | 0.0030 (0.0007, 0.0053) 0.012067 | -0.0038 (-0.0059, -0.0017) 0.000483 | -0.0001 (-0.0040, 0.0037) 0.954115 |
| Model 2 | | -0.0099 (-0.0134, -0.0063) <0.000001 | -0.0082 (-0.0121, -0.0043) 0.000045 | -0.0086 (-0.0154, -0.0018) 0.016057 |
| **Femoral neck BMD** | |  |  |  |
| Model 1 | | 0.0031 (0.0010, 0.0051) 0.003224 | -0.0052 (-0.0073, -0.0030) 0.000003 | -0.0005 (-0.0039, 0.0030) 0.785799 |
| Model 2 | | -0.0088 (-0.0122, -0.0054) <0.000001 | -0.0067 (-0.0106, -0.0028) 0.000787 | -0.0075 (-0.0140, -0.0009) 0.028887 |
| **Intertrochante BMD** | |  |  |  |
| Model 1 | | 0.0036 (0.0008, 0.0063) 0.012736 | -0.0044 (-0.0068, -0.0020) 0.000424 | -0.0001 (-0.0047, 0.0044) 0.954171 |
| Model 2 | | -0.0112 (-0.0153, -0.0071) <0.000001 | -0.0094 (-0.0140, -0.0049) 0.000062 | -0.0095 (-0.0174, -0.0016) 0.020631 |
| **Lumbar spine BMD** | |  |  |  |
| Model 1 | | 0.0023 (-0.0003, 0.0048) 0.081366 | -0.0045 (-0.0068, -0.0023) 0.000104 | -0.0048 (-0.0104, 0.0009) 0.099670 |
| Model 2 | | -0.0104 (-0.0136, -0.0072) <0.000001 | -0.0078 (-0.0121, -0.0036) 0.000372 | -0.0170 (-0.0275, -0.0065) 0.002144 |
| **Lumbar Pelvis BMD** | |  |  |  |
| Model 1 | | 0.0071 (0.0043, 0.0099) 0.000001 | -0.0009 (-0.0035, 0.0017) 0.490248 | -0.0004 (-0.0058, 0.0050) 0.886483 |
| Model 2 | | -0.0088 (-0.0129, -0.0047) 0.000034 | -0.0046 (-0.0094, 0.0002) 0.063481 | -0.0165 (-0.0262, -0.0068) 0.001363 |
| **BMI_GROUP= 3** | |  |  |  |
| **Total body BMD** | |  |  |  |
| Model 1 | | 0.0010 (-0.0006, 0.0026) 0.219561 | -0.0005 (-0.0012, 0.0003) 0.208995 | -0.0004 (-0.0017, 0.0008) 0.492446 |
| Model 2 | | -0.0072 (-0.0095, -0.0049) <0.000001 | -0.0042 (-0.0059, -0.0026) 0.000001 | -0.0052 (-0.0078, -0.0025) 0.000170 |
| **Total femur BMD** | |  |  |  |
| Model 1 | | 0.0022 (0.0004, 0.0039) 0.016429 | 0.0013 (0.0003, 0.0022) 0.007807 | 0.0018 (0.0003, 0.0033) 0.019552 |
| Model 2 | | -0.0077 (-0.0107, -0.0048) <0.000001 | -0.0044 (-0.0066, -0.0023) 0.000062 | -0.0054 (-0.0087, -0.0021) 0.001475 |
| **Femoral neck BMD** | |  |  |  |
| Model 1 | | 0.0023 (0.0008, 0.0039) 0.003168 | 0.0007 (-0.0003, 0.0017) 0.181860 | 0.0015 (0.0002, 0.0029) 0.029374 |
| Model 2 | | -0.0073 (-0.0102, -0.0043) 0.000002 | -0.0034 (-0.0056, -0.0012) 0.002678 | -0.0040 (-0.0071, -0.0009) 0.012047 |
| **Intertrochante BMD** | |  |  |  |
| Model 1 | | 0.0024 (0.0003, 0.0045) 0.023422 | 0.0015 (0.0004, 0.0025) 0.007201 | 0.0024 (0.0007, 0.0041) 0.006752 |
| Model 2 | | -0.0074 (-0.0108, -0.0039) 0.000041 | -0.0045 (-0.0070, -0.0020) 0.000493 | -0.0054 (-0.0093, -0.0015) 0.006748 |
| **Lumbar spine BMD** | |  |  |  |
| Model 1 | | 0.0004 (-0.0015, 0.0023) 0.680246 | -0.0008 (-0.0019, 0.0002) 0.124168 | 0.0001 (-0.0017, 0.0019) 0.904912 |
| Model 2 | | -0.0084 (-0.0114, -0.0055) <0.000001 | -0.0043 (-0.0069, -0.0018) 0.000835 | -0.0058 (-0.0099, -0.0017) 0.006047 |
| **Lumbar Pelvis BMD** | |  |  |  |
| Model 1 | | 0.0036 (0.0013, 0.0060) 0.002475 | 0.0018 (0.0004, 0.0031) 0.008785 | 0.0009 (-0.0010, 0.0028) 0.354716 |
| Model 2 | | -0.0089 (-0.0125, -0.0052) 0.000003 | -0.0031 (-0.0062, 0.0000) 0.051727 | -0.0052 (-0.0093, -0.0010) 0.015539 |
| **Female** | |  |  |  |
| **BMI_GROUP= 1** | |  |  |  |
| **Total body BMD** |  |  |  | |
| Model 1 | 0.0102 (0.0084, 0.0121) <0.000001 | 0.0000 (-0.0091, 0.0091) 0.997616 | NA | |
| Model 2 | -0.0027 (-0.0087, 0.0034) 0.388989 | -0.0195 (-0.0329, -0.0060) 0.046913 |  | |
| **Total femur BMD** |  |  |  | |
| Model 1 | 0.0089 (0.0071, 0.0108) <0.000001 | 0.0083 (-0.0019, 0.0185) 0.123595 | NA | |
| Model 2 | -0.0015 (-0.0091, 0.0062) 0.706979 | -0.0162 (-0.0281, -0.0043) 0.055541 |  | |
| **Femoral neck BMD** |  |  |  | |
| Model 1 | 0.0076 (0.0058, 0.0093) <0.000001 | 0.0073 (-0.0045, 0.0191) 0.236817 | NA | |
| Model 2 | 0.0004 (-0.0072, 0.0080) 0.914807 | -0.0070 (-0.0244, 0.0104) 0.476027 |  | |
| **Intertrochante BMD** |  |  |  | |
| Model 1 | 0.0113 (0.0091, 0.0134) <0.000001 | 0.0120 (0.0004, 0.0236) 0.052160 | NA | |
| Model 2 | 0.0001 (-0.0087, 0.0090) 0.977206 | -0.0207 (-0.0388, -0.0027) 0.087693 |  | |
| **Lumbar spine BMD** |  |  |  | |
| Model 1 | 0.0113 (0.0088, 0.0138) <0.000001 | -0.0031 (-0.0199, 0.0136) 0.718763 | NA | |
| Model 2 | -0.0016 (-0.0100, 0.0068) 0.715636 | -0.0328 (-0.0617, -0.0038) 0.091079 |  | |
| **Lumbar Pelvis BMD** |  |  |  | |
| Model 1 | 0.0165 (0.0139, 0.0190) <0.000001 | 0.0004 (-0.0126, 0.0133) 0.953382 | NA | |
| Model 2 | -0.0008 (-0.0107, 0.0092) 0.883256 | 0.0012 (-0.0280, 0.0304) 0.939515 |  | |
| **BMI_GROUP= 2** |  |  |  | |
| **Total body BMD** |  |  |  | |
| Model 1 | 0.0024 (0.0007, 0.0040) 0.004620 | 0.0004 (-0.0011, 0.0019) 0.558115 | -0.0026 (-0.0053, -0.0000) 0.051391 | |
| Model 2 | -0.0022 (-0.0043, -0.0002) 0.034657 | -0.0017 (-0.0038, 0.0003) 0.093224 | -0.0032 (-0.0066, 0.0003) 0.073763 | |
| **Total femur BMD** |  |  |  | |
| Model 1 | 0.0039 (0.0021, 0.0058) 0.000023 | -0.0004 (-0.0022, 0.0013) 0.625691 | -0.0004 (-0.0033, 0.0026) 0.803365 | |
| Model 2 | -0.0021 (-0.0049, 0.0006) 0.128156 | -0.0028 (-0.0052, -0.0004) 0.022019 | -0.0012 (-0.0050, 0.0026) 0.541710 | |
| **Femoral neck BMD** |  |  |  | |
| Model 1 | 0.0038 (0.0021, 0.0054) 0.000008 | -0.0006 (-0.0023, 0.0012) 0.515439 | 0.0005 (-0.0021, 0.0032) 0.692439 | |
| Model 2 | -0.0007 (-0.0034, 0.0021) 0.633828 | -0.0019 (-0.0042, 0.0004) 0.110462 | 0.0001 (-0.0035, 0.0037) 0.946729 | |
| **Intertrochante BMD** |  |  |  | |
| Model 1 | 0.0047 (0.0025, 0.0069) 0.000027 | -0.0005 (-0.0026, 0.0016) 0.632732 | 0.0002 (-0.0035, 0.0038) 0.927364 | |
| Model 2 | -0.0026 (-0.0058, 0.0006) 0.117904 | -0.0031 (-0.0060, -0.0003) 0.032293 | -0.0012 (-0.0059, 0.0036) 0.637394 | |
| **Lumbar spine BMD** |  |  |  | |
| Model 1 | 0.0020 (-0.0002, 0.0043) 0.080678 | -0.0005 (-0.0027, 0.0016) 0.625327 | -0.0046 (-0.0085, -0.0007) 0.021485 | |
| Model 2 | -0.0061 (-0.0089, -0.0033) 0.000021 | -0.0029 (-0.0059, 0.0001) 0.058056 | -0.0027 (-0.0082, 0.0027) 0.331612 | |
| **Lumbar Pelvis BMD** |  |  |  | |
| Model 1 | 0.0065 (0.0042, 0.0088) <0.000001 | 0.0041 (0.0018, 0.0064) 0.000664 | -0.0007 (-0.0043, 0.0030) 0.714383 | |
| Model 2 | -0.0014 (-0.0046, 0.0019) 0.412382 | 0.0001 (-0.0032, 0.0034) 0.958834 | -0.0005 (-0.0055, 0.0046) 0.854260 | |
| **BMI_GROUP= 3** |  |  |  | |
| **Total body BMD** |  |  |  | |
| Model 1 | 0.0021 (0.0009, 0.0033) 0.000592 | 0.0002 (-0.0005, 0.0009) 0.537293 | 0.0007 (-0.0005, 0.0018) 0.274127 | |
| Model 2 | -0.0024 (-0.0039, -0.0009) 0.002245 | -0.0021 (-0.0035, -0.0008) 0.001924 | -0.0023 (-0.0045, -0.0002) 0.035721 | |
| **Total femur BMD** |  |  |  | |
| Model 1 | 0.0040 (0.0027, 0.0054) <0.000001 | 0.0027 (0.0018, 0.0036) <0.000001 | 0.0023 (0.0009, 0.0037) 0.001735 | |
| Model 2 | -0.0026 (-0.0049, -0.0003) 0.024611 | -0.0016 (-0.0034, 0.0002) 0.074418 | -0.0020 (-0.0047, 0.0007) 0.150976 | |
| **Femoral neck BMD** |  |  |  | |
| Model 1 | 0.0041 (0.0028, 0.0054) <0.000001 | 0.0026 (0.0017, 0.0035) <0.000001 | 0.0016 (0.0003, 0.0029) 0.019315 | |
| Model 2 | -0.0025 (-0.0048, -0.0001) 0.040159 | -0.0013 (-0.0030, 0.0005) 0.155424 | -0.0036 (-0.0061, -0.0011) 0.005371 | |
| **Intertrochante BMD** |  |  |  | |
| Model 1 | 0.0045 (0.0029, 0.0061) <0.000001 | 0.0028 (0.0018, 0.0039) <0.000001 | 0.0026 (0.0009, 0.0043) 0.002824 | |
| Model 2 | -0.0028 (-0.0054, -0.0002) 0.034200 | -0.0020 (-0.0041, 0.0001) 0.056594 | -0.0020 (-0.0052, 0.0013) 0.235977 | |
| **Lumbar spine BMD** |  |  |  | |
| Model 1 | 0.0013 (-0.0003, 0.0029) 0.112205 | -0.0011 (-0.0022, -0.0001) 0.032162 | 0.0016 (-0.0001, 0.0034) 0.066094 | |
| Model 2 | -0.0035 (-0.0058, -0.0012) 0.003062 | -0.0044 (-0.0064, -0.0024) 0.000017 | -0.0019 (-0.0052, 0.0014) 0.268193 | |
| **Lumbar Pelvis BMD** |  |  |  | |
| Model 1 | 0.0046 (0.0028, 0.0063) <0.000001 | 0.0024 (0.0012, 0.0035) 0.000052 | 0.0007 (-0.0010, 0.0024) 0.403567 | |
| Model 2 | -0.0009 (-0.0038, 0.0020) 0.548318 | 0.0002 (-0.0020, 0.0025) 0.826753 | -0.0043 (-0.0074, -0.0011) 0.008851 | |

All the results were showed by β (95%CI) and p.

BMD: body mineral density. CI: confidence interval. ALP: alkaline phosphatase. UA: uric acid.

Model 1: adjusted for none.

Model 2: adjusted for age, race, poverty income ratio, height, weight, ALP, total calcium, creatinine, fasting glucose, UA and parathyroid hormone in group 1.

Model 2: adjusted for age, race, poverty income ratio, height, weight, smoking status, alcohol use, physical activity, ALP, total calcium, creatinine, fasting glucose, UA and parathyroid hormone in group 2 and group 3.
